# Factors influencing choices of empirical antibiotic treatment for bacterial infections in a scenario-based survey in Vietnam

**DOI:** 10.1093/jacamr/dlaa087

**Published:** 2020-11-10

**Authors:** Thi Lan Huong Vu, Quoc Dat Vu, Bao Long Hoang, Thi Cam Tu Nguyen, Thi Dieu Ngan Ta, Behzad Nadjm, H Rogier van Doorn

**Affiliations:** Oxford University Clinical Research Unit, 78 Giai Phong, Hanoi, Vietnam; Hanoi Medical University, 1 Ton That Tung, Hanoi, Vietnam; National Hospital for Tropical Diseases, 78 Giai Phong, Hanoi, Vietnam; Oxford University Clinical Research Unit, 78 Giai Phong, Hanoi, Vietnam; Oxford University Clinical Research Unit, 78 Giai Phong, Hanoi, Vietnam; Hanoi Medical University, 1 Ton That Tung, Hanoi, Vietnam; National Hospital for Tropical Diseases, 78 Giai Phong, Hanoi, Vietnam; Oxford University Clinical Research Unit, 78 Giai Phong, Hanoi, Vietnam; MRC Unit The Gambia at The London School of Hygiene & Tropical Medicine, Fajara, The Gambia; Oxford University Clinical Research Unit, 78 Giai Phong, Hanoi, Vietnam; Centre for Tropical Medicine and Global Health, Nuffield Department of Medicine, University of Oxford, Oxford, UK

## Abstract

**Background:**

Antimicrobial stewardship (AMS) programmes have been implemented around the world to guide rational use of antibiotics but implementation is challenging, particularly in low- and middle-income countries, including Vietnam. Understanding factors influencing doctors’ prescribing choices for empirical treatment can help design AMS interventions in these settings.

**Objectives:**

To understand doctors’ choices of antibiotics for empirical treatment of common bacterial infections and the factors influencing decision-making.

**Methods:**

We conducted a cross-sectional survey among medical professionals applying for a postgraduate programme at Hanoi Medical University, Vietnam. We used a published survey developed for internal medicine doctors in Canada. The survey was self-administered and included four clinical scenarios: (i) severe undifferentiated sepsis; (ii) mild undifferentiated sepsis; (iii) severe genitourinary infection; and (iv) mild genitourinary infection.

**Results:**

A total of 1011/1280 (79%), 683/1188 (57.5%), 718/1157 (62.1%) and 542/1062 (51.0%) of the participants selected combination therapy for empirical treatment in scenarios 1, 2, 3 and 4, respectively. Undifferentiated sepsis (OR 1.82, 95% CI 1.46–2.27 and 2.18, 1.51–3.16 compared with genitourinary) and severe infection (1.33, 1.24–1.43 and 1.38, 1.21–1.58 compared with mild) increased the likelihood of choosing a combination therapy and a carbapenem regimen, respectively. Participants with higher acceptable minimum threshold for treatment coverage and young age were also more likely to prescribe carbapenems.

**Conclusions:**

Decision-making in antibiotic prescribing among doctors in Vietnam is influenced by both disease-related characteristics and individual factors, including acceptable minimum treatment coverage. These findings are useful for tailoring AMS implementation in Vietnam and other, similar settings.

## Introduction

Antibiotic use is a selective driver for the emergence of drug-resistant organisms.^1^ The dilemma of antibiotic use,^2^ commonly termed ‘the tragedy of the commons’,^3–7^ is that both appropriate and inappropriate use of antibiotics promotes resistance. Antibiotic prescriptions have been shown to be associated with increased risks of resistance in individual patients in a systematic review and analysis.^8^ While investment in development of new antibiotics and in infection control can help reduce the impact of resistance, restrictions on antibiotic use still play a major role.^4^ A balance between immediate and individual gain versus longer-term community benefit should govern decision-making in antibiotic treatment. It is argued that asking doctors to not prescribe antibiotics in the conditions that do not need antibiotics does not raise any ethical issues. However, an ethical dilemma arises in restricting therapeutically justified antibiotic use where individual need to treat infections is judged against the collective need to preserve antibiotic effectiveness.^7^ Finding the right balance requires a better understanding of the effects that antibiotic use have on individual patients and on society as a whole.^4^

Antimicrobial stewardship (AMS) has been implemented as an essential strategy in response to the rising antibiotic use in hospitals around the world, and is often directed at restricting unjustifiable antibiotic use. Despite increasing evidence of AMS impact, decision-making on antibiotic use remains suboptimal.^9^ Globally, approximately half of patients in hospitals received inappropriate antibiotic treatment.^10^ Broad-spectrum antibiotics are attractive choices for doctors especially under conditions of uncertainty about source of infection and lack of microbiological evidence.^11^ Antibiotic prescribing in hospitals is, in simple terms, governed by availability of recent locally relevant surveillance data, prescription guidelines and authorization procedures. Studies conducted on antibiotic prescribing practices in hospitals, however, have been mainly in high-income countries.

An early review of qualitative literature showed the complexity of behaviour change in prescribing practices influenced by mutually dependent intrinsic (knowledge and attitudes) and extrinsic (patient- and healthcare system-related) factors.^12^ With a focus on risk perceptions, a recent review^11^ demonstrated that prescribers are over-reliant on antibiotics and broad-spectrum antibiotics for some perceived short-term benefits, such as quick recovery, low cost, low risk and low cognitive effort in decision-making. Although being aware of the increasing resistance problem, the argument commonly made by prescribers was that the main causes lie beyond hospital settings, including the unregulated antibiotic use in the community and the quality of drugs, and therefore curbing antibiotic prescribing in hospitals would be ineffective. This psychology of externalizing responsibility may hamper the efforts to change behaviour in hospital settings to address the antibiotic resistance problem.^11^

In addition, limited research from low- and middle-income countries (LMICs) highlighted the perception that, because of poor hygiene and infection control in these settings, doctors felt the need to prescribe broad-spectrum antibiotics for infection prevention.^13^^,^^14^ A recent review also demonstrated that interventions combining different strategies in LMICs were more effective in changing behaviour prescriber and that future interventions should address the influence of structural and contextual factors on individuals’ behaviour.^15^ More data are therefore needed to gain in-depth understanding of these dimensions to help design appropriate interventions to promote optimal antibiotic prescribing practices in hospitals in LMIC settings.

In Vietnam, by the end of 2018 AMS programmes had been initiated in 47% of hospitals^16^ since the issuance of the guideline for implementation of AMS in hospitals (Decision 772) in March 2016.^17^ However, implementation has been slow and limited to guideline development and pre-prescription authorization. Our initial qualitative observations in Vietnamese hospitals have shown that, despite doctors being aware of the issues with drug resistance, this awareness does not affect their decision-making in hospital settings.

A recent survey among internal medicine doctors in Canada used four common bacterial infections to elicit doctor’s psychology in making decisions on empirical antibiotic treatment.^18^ In this study, we used the same survey form to assess the choices of empirical antibiotic regimens and the underlying factors associated with these choices. We hypothesize that choices of empirical regimens are driven not only by doctors’ perceptions of antibiotic coverage but also by other individual and environmental factors. The results of this survey will provide more understanding of the considerations that doctors make in choosing antibiotics with adequate coverage for the range of possible causative pathogens while minimizing the use of broad-spectrum treatment, and support AMS interventions to improve antibiotic prescribing practices in Vietnam and other, similar settings.

## Methods

This study was a cross-sectional survey seeking responses from medical professionals applying for a postgraduate specialization programme in July 2019 at Hanoi Medical University, the leading medical university that offers the largest programme of postgraduate medical training in Vietnam. The survey form was adapted from a study of internal medicine doctors in Canada.^18^ In our study, we designed a tablet survey form that was handed to participants when they visited the university for the entrance examination mock test. Participants were assured that their information was kept confidential and did not affect their examination results, and they were free to stop the survey at any time or not to answer any questions. Any subsequent data retrieval from the central database was handled in a de-identified manner.

The study was approved by the Ethics Committee for Biomedical Research of the National Hospital for Tropical Diseases (No. 07/HDDD-NDTU 30/04/2018) and the Oxford Tropical Research Ethics Committee, University of Oxford (OxTREC Reference: 520-18).

### Survey questions, adaptation and translation

The original survey form in English was adapted for the Vietnamese setting and translated into Vietnamese. The form was then piloted among five infectious disease specialists at the National Hospital for Tropical Diseases for content validity and any issues with the questions and the survey flow. Based on the responses, the Vietnamese survey was finalized and back-translated into English to ensure comparability with the original survey form (see Supplementary Methods, available as Supplementary data at *JAC-AMR* Online).

The survey form consists of four given sepsis scenarios with questions for participants on their choice of antibiotic regimens, their estimate of the likelihood that the selected regimen would cover the causative pathogen (perceived coverage), and the minimum threshold of coverage that the participants would be willing to accept (acceptable minimum threshold). The four sepsis scenarios varied by source of infection (undifferentiated or genitourinary) and the level of severity based on the Quick Sepsis Related Organ Failure Assessment (qSOFA) score (severe with a qSOFA score of 3 or mild with a qSOFA score of 0). Both undifferentiated and genitourinary sources of systemic infection are common clinical presentations in Vietnamese hospitals.^19–21^

A list of antibiotic options was presented for participants to choose for empirical treatment in each of the scenarios; participants could choose monotherapy or combination therapy from a list of antibiotic agents (see Supplementary Methods). Participants were asked to provide information on their clinical experience (number of years working in clinical practice), departments where they are currently working, and a self-reported estimate of their level of antibiotic prescribing compared with their peers (less, equal, more). We also extracted information on age, gender and types of training programme (residency, masters, specialized level 1 and specialized level 2) from the candidate registry at the university. Specialized level 1 training is a 2 year clinical training track for registered professionals with at least 18 months of practice in hospitals and specialized level 2 is a 2 year clinical training programme that requires applicants hold the specialized level 1 or equivalent degree. Masters training is under the 2 year academic track and residency programme is a 3 year clinical training for only newly graduated medical students.^22^^,^^23^

In addition, participants were asked how each of a number of predefined clinical and microbiological factors would affect their choice of antibiotics with respect to the breadth of its spectrum of activity. The responses were on a five-point Likert scale, the most widely used approach to scaling survey responses, asking participants to specify their opinions on a symmetrical agree–disagree scale.^24^ These factors were patient factors (age, medical comorbidity, severity of illness, residence location and previous hospital admission history) and local microbiological data (isolation of resistant organism from previous patients known to the responding clinician, report of resistant organism in surveillance swab and higher rates of resistance in local antibiogram).

### Statistical analysis

Since responses on choice of antibiotics, perceived coverage and minimum threshold are likely correlated and clustered by each participant for the four scenarios, we used the generalized estimating equation (GEE) method to fit a generalized linear model to account for non-dependence between responses within each participant. For each outcome variable, we first calculated unadjusted ORs for binary outcomes or mean difference for continuous outcomes and 95% CI in a univariate model (see Supplementary data for the unadjusted estimates). Then we ran multivariate models to derive the adjusted estimates with all hypothesized predictors, including age (years), gender (male; female), duration of clinical experience (years), infection source (genitourinary; undifferentiated), severity (mild; severe), specialty [infectious disease/ICU/emergency department (ID/ICU/ED); internal medicine; other clinical departments, which mainly includes surgery, paediatrics, obstetrics/gynaecology], type of study programme (residence; masters; specialized level 1; specialized level 2) and self-reported level of prescribing (equal to peers; less than peers; more than peers). For choice of antibiotics, we developed separate models for choice of a combination therapy and choice of carbapenems. In these models, perceived coverage (<80%; ≥80%) and minimum threshold (≤70%; >70%) were added in the predictor list as binary independent variables (the cut-off point was determined by the median). For the choice of carbapenems, choice of a combination therapy (no; yes) was also added as an independent variable.

All statistical analyses were performed in R version 3.3.3 (Foundation for Statistical Computing, Vienna, Austria). We used the geepack package within the R programme for GEE analysis.^25^

## Results

### Participant characteristics

**Figure 1.**
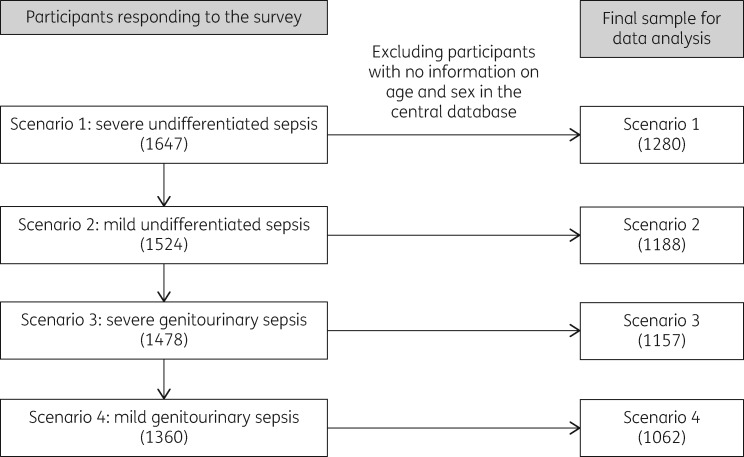
Participants responding to the scenario-based survey at Hanoi Medical University, Vietnam and the number of participants included in the final analysis.

After removing those without information on age and sex, 1280, 1188, 1157 and 1062 participants were included in the analysis for scenarios 1, 2, 3 and 4, respectively (Figure 1).

Demographic characteristics of participants were similar across the groups who provided an answer to each of the four scenarios and the group that answered all four scenarios (Table 1). Participants had a median age of 30 years with a median length of clinical experience of 4 years. Twenty percent worked in internal medicine and 12%–13% in infectious diseases, ICU and emergency. Forty-five percent to 48% applied for a specialized level 1 training programme.

**Table 1.** Demographic characteristics of the survey participants included in the analysis for four clinical scenarios: 1 (severe undifferentiated sepsis); 2 (mild undifferentiated sepsis); 3 (severe genitourinary infection); and 4 (mild genitourinary infection), and those who answered all four scenarios

	Scenario 1	Scenario 2	Scenario 3	Scenario 4	All four scenarios
Gender and age	*n* = 1280	*n* = 1188	*n* = 1157	*n* = 1062	*n* = 984
male, *n* (%)	677 (52.9)	635 (53.5)	612 (52.9)	553 (52.1)	522 (53.0)
age, median (min–max)	30 (23–54)	30 (23–54)	30 (23–54)	30 (23–50)	30 (23–49)
Years of clinical experience	*n* = 816	*n* = 768	*n* = 767	*n* = 747	*n* = 705
median (min–max)	4 (0–23)	4 (0–23)	4 (0–23)	4 (0–23)	4 (0–23)
Specialty, *n* (%)	*n* = 932	*n* = 875	*n* = 868	*n* = 848	*n* = 779
infectious diseases	26 (2.8)	23 (2.6)	22 (2.5)	26 (3.1)	21 (2.7)
intensive care	53 (5.7)	48 (5.5)	49 (5.6)	50 (5.9)	44 (5.6)
emergency	40 (4.3)	38 (4.3)	35 (4.0)	33 (3.9)	31 (4.0)
internal medicine	187 (20.1)	179 (20.5)	176 (20.3)	173 (20.4)	173 (22.2)
other clinical	487 (52.3)	457 (52.2)	453 (52.2)	446 (52.6)	398 (51.1)
other	139 (14.9)	130 (14.9)	133 (15.3)	120 (14.2)	112 (14.4)
Type of study programme, *n* (%)	*n* = 1280	*n* = 1188	*n* = 1157	*n* = 1062	*n* = 984
residency	175 (13.7)	169 (14.2)	176 (15.2)	164 (15.4)	160 (16.3)
masters	314 (24.5)	295 (24.8)	291 (25.2)	259 (24.4)	246 (25.0)
specialized level 1	610 (47.7)	553 (46.5)	517 (44.7)	479 (45.1)	426 (43.3)
specialized level 2	181 (14.1)	171 (14.4)	173 (15.0)	160 (15.1)	152 (15.4)

Data are restricted to those with information on gender and age.

### Antibiotic choices, perceived coverage and acceptable threshold for coverage of empirical treatment

More participants selected a combination therapy in severe sepsis than in mild sepsis: 1011/1280 (79%), 718/1157 (62.1%), 683/1188 (57.5%) and 542/1062 (51.0%) for severe undifferentiated, severe genitourinary, mild undifferentiated and mild genitourinary sepsis scenarios, respectively (Table 2). Most participants considered their level of antibiotic prescribing frequency as equal to (52%) or less than (37%) other doctors in their field. Similar results were observed in the participants who answered all four scenarios (Table S1).

**Table 2.** Antibiotic prescribing practices among the surveyed participants in the four clinical scenarios: 1 (severe undifferentiated sepsis); 2 (mild undifferentiated sepsis); 3 (severe genitourinary infection); and 4 (mild genitourinary infection)

	Scenario 1	Scenario 2	Scenario 3	Scenario 4
Choice of empirical antibiotic treatment	*n* = 1280	*n* = 1188	*n* = 1157	*n* = 1062
one antibiotic	269 (21.0)	505 (42.5)	439 (37.9)	520 (49.0)
more than one antibiotic	1011 (79.0)	683 (57.5)	718 (62.1)	542 (51.0)
Perceived coverage of empirical antibiotic treatment choice^a^	*n* = 610	*n* = 524	*n* = 517	*n* = 493
coverage ≥80%	370 (60.7)	282 (53.8)	336 (65.0)	305 (61.9)
coverage <80%	240 (39.3)	242 (46.2)	181 (35.0)	188 (38.1)
Acceptable minimum threshold for coverage^a^	*n* = 442	*n* = 398	*n* = 402	*n* = 403
threshold >70%	194 (43.9)	160 (40.2)	197 (49.0)	194 (48.1)
threshold ≤70%	248 (56.1)	238 (59.8)	205 (51.0)	209 (51.9)
Self-reported prescribing (relative to peers)	*n* = 851	*n* = 800	*n* = 792	*n* = 779
equal	441 (51.8)	420 (52.5)	415 (52.4)	404 (51.9)
less	319 (37.5)	296 (37.0)	294 (37.1)	293 (37.6)
more	91 (10.7)	84 (10.5)	83 (10.5)	82 (10.5)

Data are restricted to those with information on gender and age. Data for those who answered all four scenarios are presented in Table S1.

a Using median as a cut-off point.

The mean perceived coverage of selected empirical therapy was lower in undifferentiated than in genitourinary scenarios: 74.4% (SD 16.7%; median 80%) in severe and 72.9% (16.7%; 80%) in mild undifferentiated sepsis, and 76.7% (15.8%; 75%) in severe and 75.2% (16.9%; 80%) in mild genitourinary infection. A coverage level of ≥80% was reported in 370/610 (60.7%), 282/524 (53.8%), 336/517 (65.0%) and 305/493 (61.9%) for severe undifferentiated, severe genitourinary, mild undifferentiated and mild genitourinary sepsis scenarios, respectively.

The mean acceptable minimum thresholds were similar in the four clinical scenarios: 69.6% (SD 16.6%; median 70%), 68.5% (16.9%; 70%), 71.1% (16.4%; 70%) and 69.9% (18.9%; 70%). A threshold of >70% for coverage was considered acceptable in 194/442 (43.9%), 160/398 (40.2%), 197/402 (49.0%) and 194/403 (48.1%) for severe undifferentiated, severe genitourinary, mild undifferentiated and mild genitourinary sepsis scenarios, respectively.

**Figure 2.**
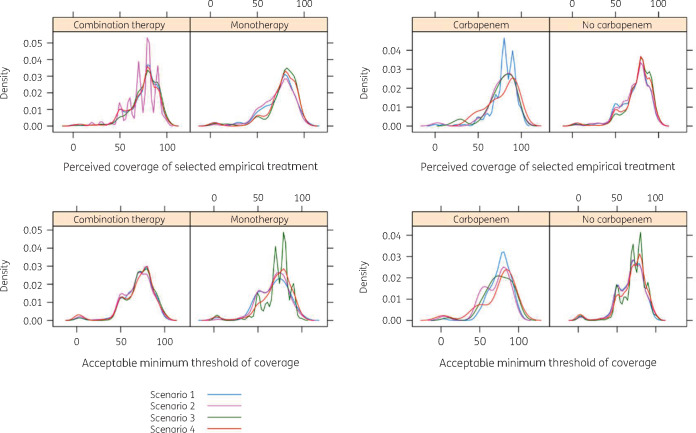
Perceived coverage of antibiotic therapy and minimum acceptable threshold of coverage among the surveyed participants in each of the clinical scenarios: scenario 1 (severe undifferentiated sepsis); 2 (mild undifferentiated sepsis); 3 (severe genitourinary infection); and 4 (mild genitourinary infection).

Perceived coverage and acceptable minimum threshold followed similar distributions, with a higher peak found for coverage across types of antibiotic regimen (Figure 2). The proportion of participants indicating a lower coverage compared with their acceptable minimum threshold was 58/435 (13.3%), 48/400 (12.0%), 62/394 (15.7%) and 55/395 (13.9%) for severe undifferentiated, severe genitourinary, mild undifferentiated and mild genitourinary sepsis scenarios, respectively. This proportion was significantly higher among those applying for the residence programme (31/160, 19.4%, *P* = 0.04, *χ*^2^ test).

**Figure 3.**
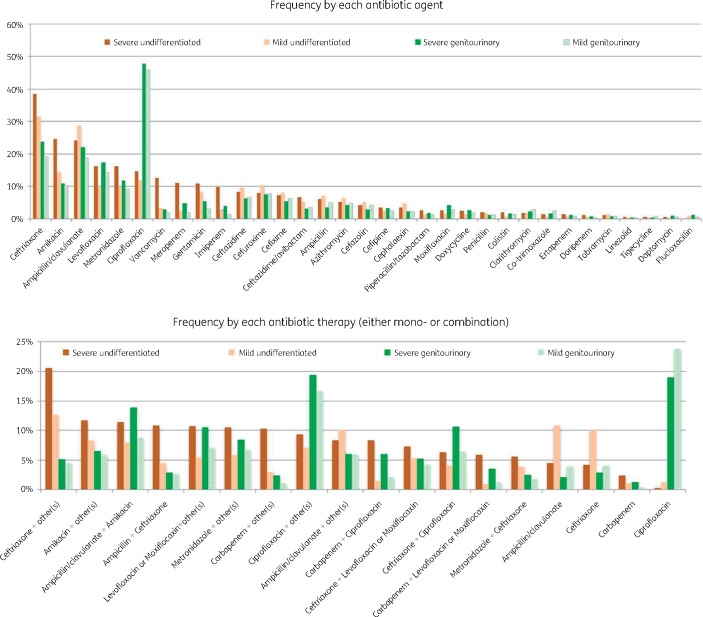
Choices of antibiotics among the surveyed participants in each of the four clinical scenarios: severe undifferentiated sepsis, mild undifferentiated sepsis, severe genitourinary infection and mild genitourinary infection.

Ceftriaxone was the most common antibiotic selected for empirical treatment in severe (37%, *n* = 1280) and mild undifferentiated sepsis (31%, *n* = 1188), and ciprofloxacin for severe (48%, *n* = 1157) and mild genitourinary sepsis (46%, *n* = 1062) (Figure 3). The most common empirical treatment regimens for severe undifferentiated sepsis were ampicillin/clavulanate + amikacin (11%), ceftriaxone+  amikacin (11%) and carbapenems + ciprofloxacin (8%). For mild undifferentiated sepsis, these were ampicillin/clavulanate monotherapy (11%), ceftriaxone monotherapy (10%) and ampicillin/clavulanate + amikacin (8%). For genitourinary sepsis, the most common treatment regimens were ciprofloxacin monotherapy, ampicillin/clavulanate + amikacin and ceftriaxone + ciprofloxacin for both severe (19%, 14% and 11%, respectively) and mild (24%, 9% and 6%, respectively) scenarios.

### Factors associated with antibiotic choices, perceived coverage and acceptable threshold for coverage of empirical treatment

Disease-related factors significantly influenced choice of combination therapy (OR 1.82, 95% CI 1.46–2.27 for undifferentiated compared with genitourinary and OR 1.33, 95% CI 1.24–1.43 for severe compared with mild infection) and choice of carbapenems (OR 2.18, 95% CI 1.51–3.16 and OR 1.38, 95% CI 1.21–1.58), respectively (Table 3). Combination therapy was also more common in those applying for a residence programme than in others (OR 3.20, 95% CI 1.09–9.40).

**Table 3.** Factors associated with choice of combination therapy over a monotherapy and choice of carbapenem versus no carbapenem therapy for empirical antibiotic treatment among the surveyed participants working in a clinical specialty

Factor	Combination versus monotherapy		Carbapenem versus no carbapenem	
	OR (95% CI)	*P* value	OR (95% CI)	*P* value
Age (per year)	1.00 (0.90–1.11)	0.99	0.86 (0.74–1.00)	0.05
Gender (female versus male)	0.88 (0.60–1.29)	0.51	0.94 (0.57–1.54)	0.79
Clinical experience (per year)	1.03 (0.91–1.15)	0.65	1.07 (0.89–1.29)	0.49
Disease-related factors				
infection source (undifferentiated versus genitourinary)	1.82 (1.46–2.27)	<0.001	2.18 (1.51–3.16)	<0.001
severity (severe versus mild)	1.33 (1.24–1.43)	<0.001	1.38 (1.21–1.58)	<0.001
Choices and perceptions				
combination therapy (versus monotherapy)			3.84 (1.90–7.74)	<0.001
Perceived coverage of empirical treatment (≥80% versus <80%)	0.91 (0.64–1.28)	0.58	1.26 (0.70–2.25)	0.44
Acceptable minimum threshold (>70% versus ≤70%)	1.18 (0.80–1.72)	0.40	2.31 (1.36–3.93)	0.002
Clinical specialty (versus ID/ICU/ED)				
internal medicine	1.06 (0.42–2.71)	0.90	0.57 (0.18–1.75)	0.32
other clinical department	0.85 (0.37–1.94)	0.70	0.61 (0.23–1.64)	0.33
Type of study programme (versus specialized level 2)				
residence	3.20 (1.09–9.40)	0.03	0.35 (0.07–1.64)	0.18
masters	1.01 (0.51–2.02)	0.97	0.50 (0.18–1.39)	0.18
specialized level 1	0.85 (0.45–1.60)	0.61	0.46 (0.18–1.17)	0.10
Self-reported prescribing intensity (versus equal to peers)				
less than peers	0.76 (0.51–1.12)	0.16	1.35 (0.82–2.23)	0.23
more than peers	0.54 (0.25–1.16)	0.12	1.76 (0.61–5.09)	0.30

Results were obtained from a multivariable GEE analysis with autoregressive correlation structure (missing data were omitted).

ORs for age and clinical experience are per year increase.

Choice of carbapenems was significantly more common in participants who chose a combination therapy (OR 3.84, 95% CI 1.90–7.74), and had a higher acceptable minimum threshold of coverage of the empirical treatment (OR 2.31, 95% CI 1.36–3.93). Those of older age tended to prescribe carbapenems less than the younger participants (OR 0.86, 95% CI 0.74–1.00 for each year increase in age).

Perceived coverage of treatment was lower in undifferentiated sepsis compared with genitourinary infection (mean difference −1.82%, 95% CI −3.02% to −0.63%) and higher in severe compared with mild infection (mean difference 0.82%, 95% CI 1.05% to 1.16%) (Table 4). Female participants were more likely to indicate a lower coverage than the male participants (mean difference −3.35%, 95% CI −6.51% to −0.19%). A lower acceptable minimum threshold for coverage was accepted in undifferentiated (mean difference −1.73%, 95% CI −2.86% to −0.60%) compared with genitourinary sepsis. However, the participants indicated a higher threshold when the condition was severe compared with mild (mean difference 0.47%, 95% CI 0.14% to 0.79%).

**Table 4.** Factors associated with perceived coverage and acceptable minimum threshold for coverage of empirical antibiotic treatment among the surveyed participants working in a clinical specialty

Factor	Perceived coverage		Acceptable minimum threshold	
	mean difference (95% CI)	*P* value	mean difference (95% CI)	*P* value
Age (per year)	−0.20 (−1.57; 1.17)	0.77	−0.35 (−1.71; 1.01)	0.61
Gender (female versus male)	−3.35 (−6.51; −0.19)	0.04	−0.63 (−4.32; 3.06)	0.74
Clinical experience (per year)	0.21 (−1.24; 1.66)	0.78	0.27 (−1.18; 1.72)	0.71
Disease-related factors				
infection source (undifferentiated versus genitourinary)	−1.82 (−3.02; −0.63)	0.003	−1.73 (−2.86; −0.60)	0.003
severity (severe versus mild)	0.82 (0.50; 1.14)	<0.001	0.47 (0.14; 0.79)	0.005
Specialty (versus ID/ICU/ED)				
internal medicine	−0.83 (−8.64; 6.98)	0.84	−5.15 (−13.91; 3.62)	0.25
other clinical department	−1.34 (−8.11; 5.43)	0.70	−4.84 (−12.31; 2.63)	0.20
Type of study programme (versus specialized level 2)				
residence	4.43 (−3.95; 12.81)	0.30	5.60 (−4.14; 15.35)	0.26
masters	−4.51 (−10.39; 1.37)	0.13	−2.85 (−9.68; 3.99)	0.41
specialized level 1	−2.36 (−7.43; 2.71)	0.36	−3.54 (−9.31; 2.23)	0.23
Self-reported prescribing intensity (versus equal to peers)				
less than peers	−0.74 (−4.22; 2.74)	0.68	−0.44 (−4.23; 3.35)	0.82
more than peers	0.15 (−4.27; 4.56)	0.95	0.13 (−6.14; 6.40)	0.97

GEE analysis with autoregressive correlation structure; missing data were omitted.

ORs for age and clinical experience are per year increase.

Analysis for the participants who answered all four scenarios gave similar results for the factors influencing choice of regimens, perceived coverage and acceptable minimum threshold for coverage of antibiotic treatment (Tables S2 and S3).

### Factors influencing doctors’ decision on antibiotic spectrum for empirical treatment

**Figure 4.**
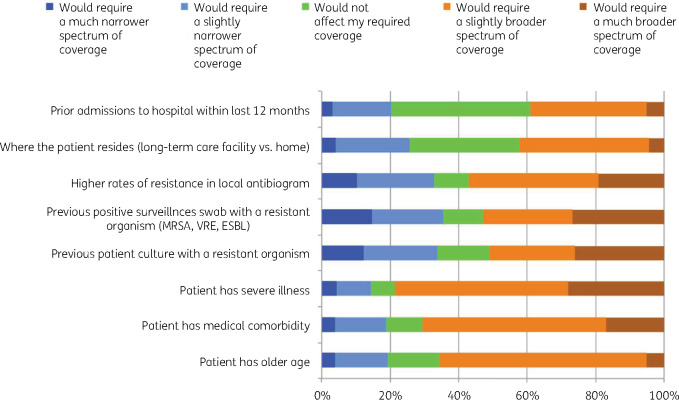
Factors influencing the decision on antibiotic spectrum for empirical treatment among the surveyed participants (*n* = 1147).

We presented eight factors and asked the participants if each of these would influence their decision to choose a regimen with narrower- or broader-spectrum antibiotics (Figure 4). Overall, participants tended to choose a broader spectrum when the patient had a more severe illness (78.6%), a higher degree of medical comorbidity (70.6%) or older age (65.7%). The responses were more symmetrically distributed for other factors presented.

## Discussion

We described factors that could influence doctors’ choice of antibiotics in empirical treatment of bacterial infections from a self-administered survey of prospective medical postgraduate students in Hanoi, Vietnam. Disease factors were found to strongly predict the choice of combination therapy and choice of carbapenems in their empirical treatment. Severity of illness and uncertainty about the source of infection play equally important roles in doctors’ decision-making on treatment regimens. These disease factors also determined the level of coverage expected and the minimum threshold of coverage accepted by the doctors in a given clinical scenario. Importantly, having a higher acceptable minimum threshold will also make doctors more likely to use carbapenems in their treatment. This information helps us understand doctors’ decision-making in the context of uncertainty, an unavoidable reality in clinical settings for which tolerance of uncertainty is needed.^26^ Identifying factors influencing this decision-making process is important in designing effective behaviour change interventions to reinforce appropriate antibiotic prescribing in hospitals.

The reported minimum threshold among our Vietnamese doctors is lower than the threshold accepted by Canadian doctors (80%–90%) in a similar survey of 237 general internists and infectious diseases specialists.^18^ This might indicate a higher tolerance of risk of treatment failure among the Vietnamese doctors compared with the Canadian peers. The absence of relevant local data, higher rates of resistance and absence of publicly available hospital indicators may be the reason for this. However, this may also be the result of frequent exposure to severe infections in settings like Vietnam, since experience in a given field allows one to foresee possible future courses and reduce uncertainty in dealing with similar situations.^27^ Clinical experience has been reported to be associated with higher tolerance of uncertainty and less risk aversion.^28^ This factor was also found to be predictive of the acceptable minimum threshold of coverage in empirical antibiotic treatment in the Canadian doctors.^18^

Interestingly, there was no evidence of association between clinical experience and doctors’ choice of treatment regimens and perceived coverage and acceptable treatment threshold. However, those applying for a residence programme were more likely to select a combination therapy; the age range of these participants was 23–27 years. In addition, younger participants were also more likely to prescribe carbapenems than their older peers. There can be an interaction between clinical experience and age in shaping doctor’s prescribing practices. However, adding an interaction term or removing these factors from the models did not change the main results of this study.

Currently, empirical treatment for sepsis worldwide is based on the ‘best-guess’ approach with broad-spectrum antibiotics to cover the likely pathogens and resistance patterns.^29^ The national treatment guideline also recommends the use of combination therapy for undifferentiated sepsis with or without risk of hospital-acquired infection (HAI); a carbapenem combination is recommended in patients with neutropenia or immunosuppression, or where HAI is likely.^30^ Carbapenems are among the restricted antibiotics that require consultation and pre-authorization before use as recommended in the national guideline for AMS implementation.^17^ In our study, participants were also more likely to select a carbapenem when they decided to use a combination therapy for empirical treatment. The overall high proportions choosing a combination therapy as well as carbapenem-containing combination therapy indicate a high level of compliance with the national guideline and might reflect the generally perceived high risk of HAIs among the Vietnamese doctors. In our survey of perceptions among 90 doctors across seven hospitals locally, 88% agreed that patients could acquire infection with an MDR organism in their institution (V.T.L. Huong, N.T.C. Tu, T.D. Ngan, B. Nadjm, N.V. Kinh and H.R. van Doorn, unpublished data). Previous studies conducted locally also reported a high incidence of HAI and colonization of MDR organisms in hospital settings in Vietnam.^31–34^ AMS educational interventions can provide more concrete guidelines to support local doctors in stratifying HAI risks in patients for appropriate empirical antibiotic treatment.

The strength of this study is its inclusion of all types of clinical departments in Vietnam as the training programmes offered were not restricted to any specific clinical discipline. This survey also has a reasonably large sample size and therefore collected responses from a number of doctors working in special settings, including infectious diseases, ICU and emergency, sufficient for multivariable analyses. The sample also covers a good representation of doctors from early to late career stage. These factors have made our study sample likely to reflect the experience of antibiotic prescribing among doctors in Vietnam.

The study did not have an issue with response rate; however, the data analyses were restricted to those with information on age and gender. We could not measure how this restriction might have caused bias in our results. Similarly, we could not assess how the fact that participants were medical professionals applying for postgraduate training and therefore might not be representative of all doctors in Vietnam might influence the overall choices of empirical treatment in this survey. In addition, a number of participants skipped or provided non-valid responses to the questions on perceived coverage and acceptable minimum threshold. These questions were complex and required participants to think more deeply into their choice of antibiotics for empirical treatment, especially when decision-making was influenced by organizational, professional and social norms pre-existing in their settings. Missing data were one limitation in our multivariable analyses for perceived coverage and minimum threshold; however, missing data were likely to be not statistically significant as the results were similar in the non-missing data for all participants and for those who answered all four scenarios. Finally, interpretation of the findings needs to take into account the fact that the four clinical scenarios presented in this survey were hypothetical and did not capture all the details of the disease trajectories that doctors might be faced with in real-world situations.

In conclusion, this study provides important insights into the decision-making process of Vietnamese doctors in empirical antibiotic treatment. Their choices of antibiotics were influenced by both disease-related factors, including severity and source of infection, and individual acceptable minimum threshold for treatment coverage. These insights can be used to guide stewardship programmes aiming at behaviour change in antibiotic prescribing practices. Such programmes should not only address the knowledge gap for individual prescribers but also consider the factors that can influence their personal perceptions of risks and decision-making under uncertainty in the clinical setting.

## Supplementary Material

dlaa087_Supplementary_Data
