# Supplementary material for: Factors influencing choices of empirical antibiotic treatment for bacterial infections in a scenario-based survey in Vietnam
Source: JAC Antimicrob Resist. 2020 Nov 10;2(4):dlaa087. doi: 10.1093/jacamr/dlaa087 (PMC7653509; doi:10.1093/jacamr/dlaa087)
Supplement: dlaa087_Supplementary_Data [file dlaa087_supplementary_data.docx]

**Supplementary data**

**Supplementary Methods**

***Back-translated version of the survey questions from Vietnamese to English***

**Scenario 1:**

A 77-year-old patient presents from home to the emergency department with a sepsis syndrome. The patient is unwell, has no localizing infectious symptoms. The patient is febrile (T max 39.2) with blood pressure of 80/50, heart rate of 100, respiratory rate of 24 and GCS of 13. Cardiorespiratory and abdominal exams are unremarkable. Complete blood count results show a leukocytosis of 24 G/L, blood lactate of 5. Blood and urine cultures are drawn. You decide this patient needs admission and you start them on empiric antibiotic therapy for a sepsis syndrome without apparent source.

1.1. What will be your empiric antibiotic regimen for this patient (this regimen can include one or multiple drugs):

| - Amikacin - Ampicillin - Ampicillin-clavulanate - Azithromycin - Cefazolin - Cefipime - Cefixime - Ceftazidime - Ceftazidime-avibactam - Ceftriaxone - Cefuroxime | - Cephalexin - Ciprofloxacin - Clarithromycin - Colistin - Daptomycin - Doxycycline - Doripenem - Ertapenem - Flucloxacillin - Gentamicin - Imipenem | - Levofloxacin - Linezolid - Meropenem - Metronidazole - Moxifloxacin - Penicillin - Piperacillin-tazobactam - Tigecycline - Tobramycin - Co-trimoxazole - Vancomycin |
| --- | --- | --- |

1.2. With the antibiotic(s) you selected for the above scenario, in your opinion what is the % likelihood that the antibiotic(s) will cover the offending pathogen(s)? ______% (between 0-100%)

1.3. In your opinion, what minimum probability of adequate coverage of the infecting
pathogen(s) is acceptable? ______% (between 0-100%)

**Scenario 2:**

A 73-year-old patient presents from home to the emergency department with a sepsis syndrome. The patient is febrile (T max 38.3) with blood pressure of 105/70, heart rate of 115, respiratory rate of 16 and GCS of 15. There are no localizing infectious symptoms or signs. Cardiorespiratory and abdominal exams are unremarkable. Complete blood count results show a leukocytosis of 19. Blood lactate of 1.5. Blood and urine cultures are drawn. You decide this patient needs admission and you start them on empiric antibiotic therapy for a sepsis syndrome without apparent source.

2.1. What will be your empiric antibiotic regimen for this patient (this regimen can include one or multiple drugs):

| - Amikacin - Ampicillin - Ampicillin-clavulanate - Azithromycin - Cefazolin - Cefipime - Cefixime - Ceftazidime - Ceftazidime-avibactam - Ceftriaxone - Cefuroxime | - Cephalexin - Ciprofloxacin - Clarithromycin - Colistin - Daptomycin - Doxycycline - Doripenem - Ertapenem - Flucloxacillin - Gentamicin - Imipenem | - Levofloxacin - Linezolid - Meropenem - Metronidazole - Moxifloxacin - Penicillin - Piperacillin-tazobactam - Tigecycline - Tobramycin - Co-trimoxazole - Vancomycin |
| --- | --- | --- |

2.2. With the antibiotic(s) you selected for the above scenario, in your opinion what is the % likelihood that the antibiotic(s) will cover the offending pathogen(s)? ______% (between 0-100%)

2.3. In your opinion, what minimum probability of adequate coverage of the infecting
pathogen(s) is acceptable? ______% (between 0-100%)

**Scenario 3:**

An 83 year-old patient presents from home to the emergency department with lower urinary tract symptoms, flank and suprapubic pain. The patient is unwell and is febrile (T max 38.6) with blood pressure of 90/60, heart rate of 110, respiratory rate of 22 and
GCS of 13. There is suprapubic and costovertebral angle tenderness on exam. Complete blood count results show a WBC count of 18. Blood lactate of 5. Urinalysis shows leukocytes, nitrites, and blood. Blood and urine cultures are drawn. You decide this patient needs admission and you start them on empiric antibiotic therapy for a genitourinary source of infection.

3.1. What will be your empiric antibiotic regimen for this patient (this regimen can include one or multiple drugs):

| - Amikacin - Ampicillin - Ampicillin-clavulanate - Azithromycin - Cefazolin - Cefipime - Cefixime - Ceftazidime - Ceftazidime-avibactam - Ceftriaxone - Cefuroxime | - Cephalexin - Ciprofloxacin - Clarithromycin - Colistin - Daptomycin - Doxycycline - Doripenem - Ertapenem - Flucloxacillin - Gentamicin - Imipenem | - Levofloxacin - Linezolid - Meropenem - Metronidazole - Moxifloxacin - Penicillin - Piperacillin-tazobactam - Tigecycline - Tobramycin - Co-trimoxazole - Vancomycin |
| --- | --- | --- |

3.2. With the antibiotic(s) you selected for the above scenario, in your opinion what is the % likelihood that the antibiotic(s) will cover the offending pathogen(s)? ______% (between 0-100%)

3.3. In your opinion, what minimum probability of adequate coverage of the infecting
pathogen(s) is acceptable? ______% (between 0-100%)

**Scenario 4:**

A 65 year-old patient presents from home to the emergency department with lower urinary tract symptoms, flank and suprapubic pain. The patient is febrile (T max 38.4) with blood pressure of 110/70, heart rate of 100, respiratory rate of 14 and GCS of 15. There is suprapubic and costovertebral angle tenderness. Complete blood count results show WBC count of 17. Blood lactate of 1.5. Urinalysis reveals leukocytes, nitrites, and blood. Blood and urine cultures are drawn. You decide this patient needs admission and you start them on empiric antibiotic therapy for a genitourinary source of infection.

4.1. What will be your empiric antibiotic regimen for this patient (this regimen can include one or multiple drugs):

| - Amikacin - Ampicillin - Ampicillin-clavulanate - Azithromycin - Cefazolin - Cefipime - Cefixime - Ceftazidime - Ceftazidime-avibactam - Ceftriaxone - Cefuroxime | - Cephalexin - Ciprofloxacin - Clarithromycin - Colistin - Daptomycin - Doxycycline - Doripenem - Ertapenem - Flucloxacillin - Gentamicin - Imipenem | - Levofloxacin - Linezolid - Meropenem - Metronidazole - Moxifloxacin - Penicillin - Piperacillin-tazobactam - Tigecycline - Tobramycin - Co-trimoxazole - Vancomycin |
| --- | --- | --- |

4.2. With the antibiotic(s) you selected for the above scenario, in your opinion what is the % likelihood that the antibiotic(s) will cover the offending pathogen(s)? ______% (between 0-100%)

4.3. In your opinion, what minimum probability of adequate coverage of the infecting
pathogen(s) is acceptable? ______% (between 0-100%)

**General information**

5. What factors would influence how broad spectrum your initial empiric
antibiotic treatment would be?

| Factor | 1 = Would require a much narrower spectrum of coverage | 2 = Would require a slightly narrower spectrum of coverage | 3 = Would not affect my required coverage | 4 = Would require a slightly broader spectrum of coverage | 5 = Would require a much broader spectrum of coverage |
| --- | --- | --- | --- | --- | --- |
| Patient has older age | ☐ | ☐ | ☐ | ☐ | ☐ |
| Patient has medical comorbidity | ☐ | ☐ | ☐ | ☐ | ☐ |
| Patient has severe illness | ☐ | ☐ | ☐ | ☐ | ☐ |
| Previous patient’s culture with a resistant organism in this hospital | ☐ | ☐ | ☐ | ☐ | ☐ |
| Previous positive surveillance swab with a resistant organism (MRSA, VRE, ESBL) in this hospital | ☐ | ☐ | ☐ | ☐ | ☐ |
| Higher rates of resistance in this hospital than in other settings | ☐ | ☐ | ☐ | ☐ | ☐ |
| Where the patient resides before admission (long-term care facility versus home) | ☐ | ☐ | ☐ | ☐ | ☐ |
| Patient’s prior admissions to hospital within last 12 months | ☐ | ☐ | ☐ | ☐ | ☐ |

6. Do you think you have a higher or lower rate of broad spectrum antibiotic
prescribing than your peers in the same clinical specialty? ☐ Higher ☐ Average ☐ Lower ☐ Other (please specify):_____________

**Table S1.** Antibiotic prescribing practices among the surveyed participants in the four clinical scenarios: 1 (severe undifferentiated sepsis); 2 (mild undifferentiated sepsis); 3 (severe genitourinary infection); 4 (mild genitourinary infection) for those who answered all 4 scenarios

|  | Scenario 1 | Scenario 2 | Scenario 3 | Scenario 4 |
| --- | --- | --- | --- | --- |
| Choice of empiric antibiotic treatment | n=984 | n=984 | n=984 | n=984 |
| One antibiotic | 195(19.8) | 400(40.7) | 358(36.4) | 481(48.9) |
| More than one antibiotic | 789(80.2) | 584(59.3) | 626(63.6) | 503(51.1) |
| Perceived coverage of empiric antibiotic treatment choice* | n=542 | n=496 | n=496 | n=474 |
| Coverage ≥80% | 333(61.4) | 269(54.2) | 322(64.9) | 293(61.8) |
| Coverage <80% | 209 (38.6) | 227(45.8) | 174(35.1) | 181(38.2) |
| Acceptable minimum threshold for coverage* | n=403 | n=380 | n=389 | n=389 |
| Threshold >70% | 180(44.7) | 156(41.1) | 191(50.9) | 186(52.2) |
| Threshold ≤70% | 223(55.3) | 224(58.9) | 198(49.1) | 203(47.8) |
| Self-reported prescribing (relative to peers) | n=724 | n=724 | n=724 | n=724 |
| Equal | 381(52.6) | 381(52.6) | 381(52.6) | 381(52.6) |
| Less | 268(37.0) | 268(37.0) | 268(37.0) | 268(37.0) |
| More | 75 (10.4) | 75 (10.4) | 75 (10.4) | 75 (10.4) |

Data is restricted to those with information on gender and age; *Using median as a cut off point.

**Table S2.** Factors associated with choice of combination therapy over a monotherapy and choice of carbapenem versus no carbapenem therapy for empiric antibiotic treatment in multivariable analysis among the surveyed participants working in a clinical specialty who answered all 4 scenarios

| Factor | Combination versus monotherapy | | Carbapenem versus no carbapenem | |
| --- | --- | --- | --- | --- |
|  | OR (95%CI) | p-value | OR (95%CI) | p-value |
| Age (per year) | 1.03(0.92-1.16) | 0.59 | 0.86(0.74-1.01) | 0.08 |
| Gender (female versus male) | 0.90(0.60-1.33) | 0.59 | 0.75(0.44-1.25) | 0.27 |
| Clinical experience (per year) | 1.03(0.90-1.17) | 0.70 | 1.04(0.86-1.26) | 0.70 |
| Disease related factors |  |  |  |  |
| Infection source (Undifferentiated versus Genitourinary) | 1.80(1.44-2.24) | <0.001 | 2.22(1.50-3.28) | <0.001 |
| Severity (severe versus mild) | 1.34(1.25-1.44) | <0.001 | 1.42(1.23-1.65) | <0.001 |
| Choices and perceptions |  |  |  |  |
| Combination therapy (versus mono-therapy) |  |  | 4.45(2.09-9.51) | <0.001 |
| Perceived coverage of empiric treatment (≥80% versus <80%) | 0.88(0.61-1.25) | 0.47 | 0.99(0.54-1.80) | 0.97 |
| Acceptable minimum threshold (>70% versus ≤70%) | 1.16(0.79-1.72) | 0.44 | 2.52(1.45-4.37) | 0.001 |
| Clinical specialty (versus ID/ICU/ED) |  |  |  |  |
| Internal Medicine | 1.05(0.40-2.75) | 0.91 | 0.56(0.18-1.77) | 0.33 |
| Other Clinical Department | 0.76(0.33-1.78) | 0.53 | 0.57(0.21-1.55) | 0.27 |
| Type of study programme (versus specialized level 2) |  |  |  |  |
| Residence | 4.55(1.53-13.5) | 0.006 | 0.28(0.05-1.43) | 0.13 |
| Masters | 1.22(0.61-2.45) | 0.57 | 0.34 (0.12-0.98) | 0.05 |
| Specialized level 1 | 1.00(0.53-1.90) | 1.00 | 0.35(0.13-0.93) | 0.04 |
| Self-reported prescribing intensity (versus Equal to peers) |  |  |  |  |
| Less than peers | 0.76(0.51-1.14) | 0.18 | 1.21(0.73-2.02) | 0.46 |
| More than peers | 0.53(0.24-1.19) | 0.12 | 1.69(0.51-5.58) | 0.39 |

Results were obtained from a multivariable GEE analysis with autoregressive correlation structure (missing data were omitted); ID/ICU/ED consists of infectious diseases, intensive care and emergency department;

**Table S3.** Factors associating with perceived coverage and acceptable minimum threshold for coverage of empiric antibiotic treatment in multivariable analysis among the surveyed participants working in a clinical specialty who answered all 4 scenarios

| Factor | Perceived coverage | | Acceptable minimum threshold | |
| --- | --- | --- | --- | --- |
|  | Mean difference (95%CI) | p-value | Mean difference (95%CI) | p-value |
| Age (per year) | 0.47(-0.35; 1.29) | 0.26 | 0.24(-0.67; 1.15) | 0.60 |
| Gender (female versus male) | -4.13(-7.35; -0.91) | 0.01 | -1.34(-5.16; 2.48) | 0.49 |
| Clinical experience (per year) | -0.48(-1.54; 0.58) | 0.37 | -0.25(-1.38; 0.88) | 0.67 |
| Disease related factors |  |  |  |  |
| Infection source (Undifferentiated versus Genitourinary) | -1.75(-2.95; -0.54) | 0.004 | -1.59(-2.74; -0.44) | 0.007 |
| Severity (severe versus mild) | 0.83(0.50; 1.15) | <0.001 | 0.47(0.14; 0.79) | 0.005 |
| Specialty (versus ID/ICU/ED) |  |  |  |  |
| Internal Medicine | 1.24(-6.34; 8.82) | 0.75 | -3.74(-12.55; 5.07) | 0.41 |
| Other Clinical Department | -0.65(-7.52; 6.22) | 0.85 | -4.71(-12.50; 3.08) | 0.24 |
| Type of study programme (versus specialized level 2) |  |  |  |  |
| Residence | 6.65(-0.74; 14.04) | 0.08 | 8.38(-0.81; 17.57) | 0.07 |
| Masters | -3.72(-9.39; 1.96) | 0.20 | -1.04(-7.89; 5.80) | 0.76 |
| Specialized level 1 | -1.24(-6.08; 3.59) | 0.36 | -2.50(-8.19; 3.20) | 0.39 |
| Self-reported prescribing intensity (versus Equal to peers) |  |  |  |  |
| Less than peers | -0.74(-4.22; 2.74) | 0.61 | -0.21(-4.10; 3.69) | 0.92 |
| More than peers | -0.42(-4.77; 3.94) | 0.85 | -0.42(-6.82; 5.99) | 0.90 |

GEE analysis with autoregressive correlation structure; missing data were omitted; ID/ICU/ED: consist of infectious disease, intensive care and emergency department.
